# PADs and NETs in digestive system: From physiology to pathology

**DOI:** 10.3389/fimmu.2023.1077041

**Published:** 2023-01-24

**Authors:** Yi-Hang Song, Zhi-Jie Wang, Le Kang, Zi-Xuan He, Sheng-Bing Zhao, Xue Fang, Zhao-Shen Li, Shu-Ling Wang, Yu Bai

**Affiliations:** Department of Gastroenterology, Changhai Hospital, Naval Medical University, Shanghai, China

**Keywords:** peptidylarginine deiminase, citrullination, digestive system, inflammation, cancer

## Abstract

Peptidylarginine deiminases (PADs) are the only enzyme class known to deiminate arginine residues into citrulline in proteins, a process known as citrullination. This is an important post-translational modification that functions in several physiological and pathological processes. Neutrophil extracellular traps (NETs) are generated by NETosis, a novel cell death in neutrophils and a double-edged sword in inflammation. Excessive activation of PADs and NETs is critically implicated in their transformation from a physiological to a pathological state. Herein, we review the physiological and pathological functions of PADs and NETs, in particular, the involvement of PAD2 and PAD4 in the digestive system, from inflammatory to oncological diseases, along with related therapeutic prospects.

## 1 Introduction

**Figure 1:**
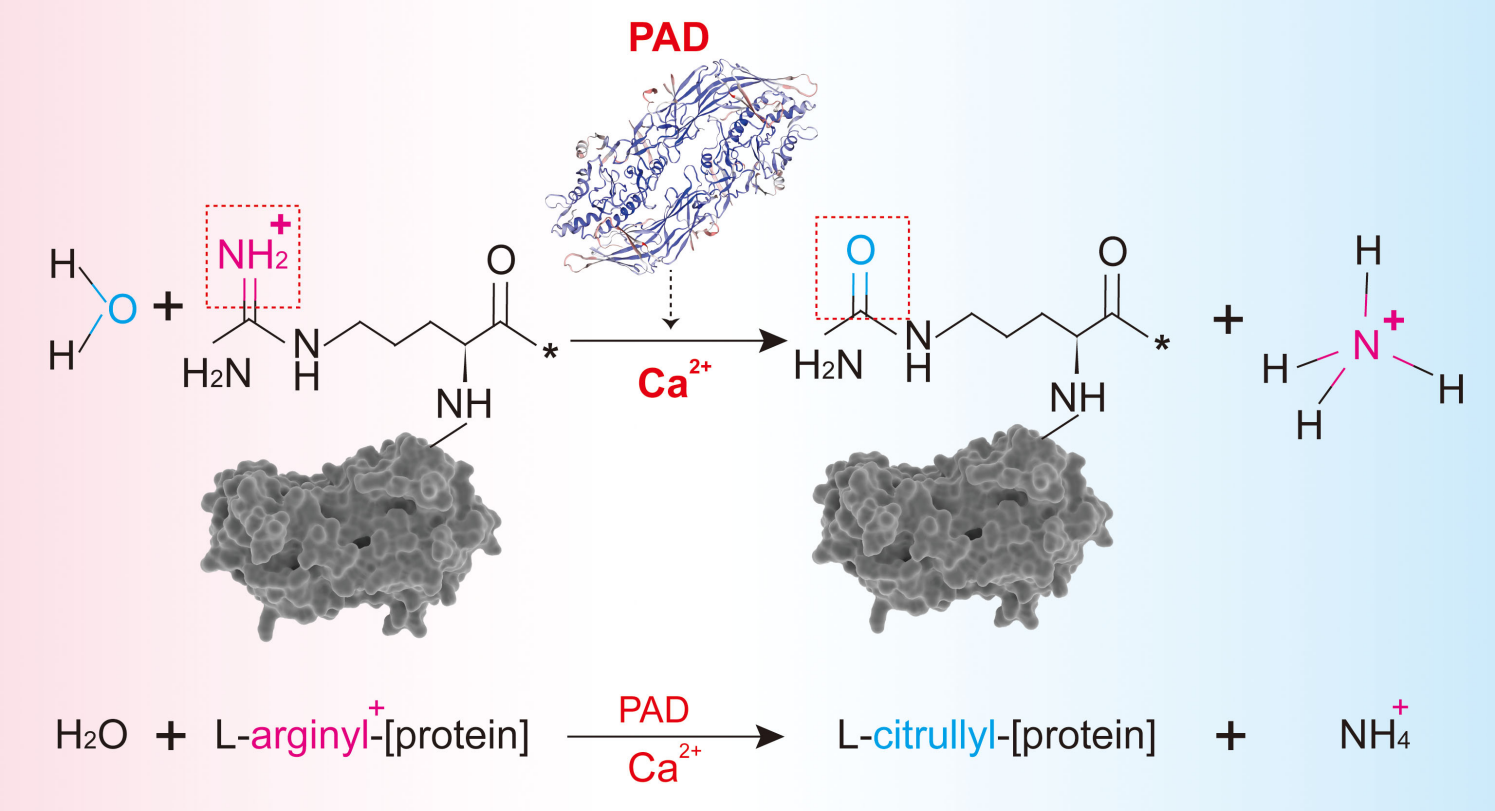
The process of citrullination mediated by PAD. **(A)** Protein citrullination mediated by PADs. **(B)** Primary substrates, distribution, and physiological functions of PADs.

Peptidylarginine deiminases (PADs), a family of homologous and conserved enzymes, are responsible for citrullination and are frequently studied. PADs occur in five isoforms (PAD 1–4 and PAD6). Interestingly, PAD5 was initially misidentified and later confirmed to be PAD4. The gene names of PADs in humans are *PADI*s (*PADI1*, *PADI2*, *PADI3*, *PADI4*, and *PADI6*). These are located on the short arm of chromosome 1 (1). As an important post-translational modification, citrullination refers to the conversion of arginine to citrulline residues in the protein-peptide chain by the catalysis of PADs (2). In the presence of calcium ions, one molecule of water reacts with a positively charged arginine residue in the protein, releasing an ammonium ion, and leading to the loss of the positive charge of the protein along with a slight mass deviation (**Figure 1**). To date, citrullination is believed to be irreversible. Changes in the electric charge alter the protein’s structure, its interaction with other proteins, and even susceptibility to degradation, together impacting its original function (3).

**Figure 2:**
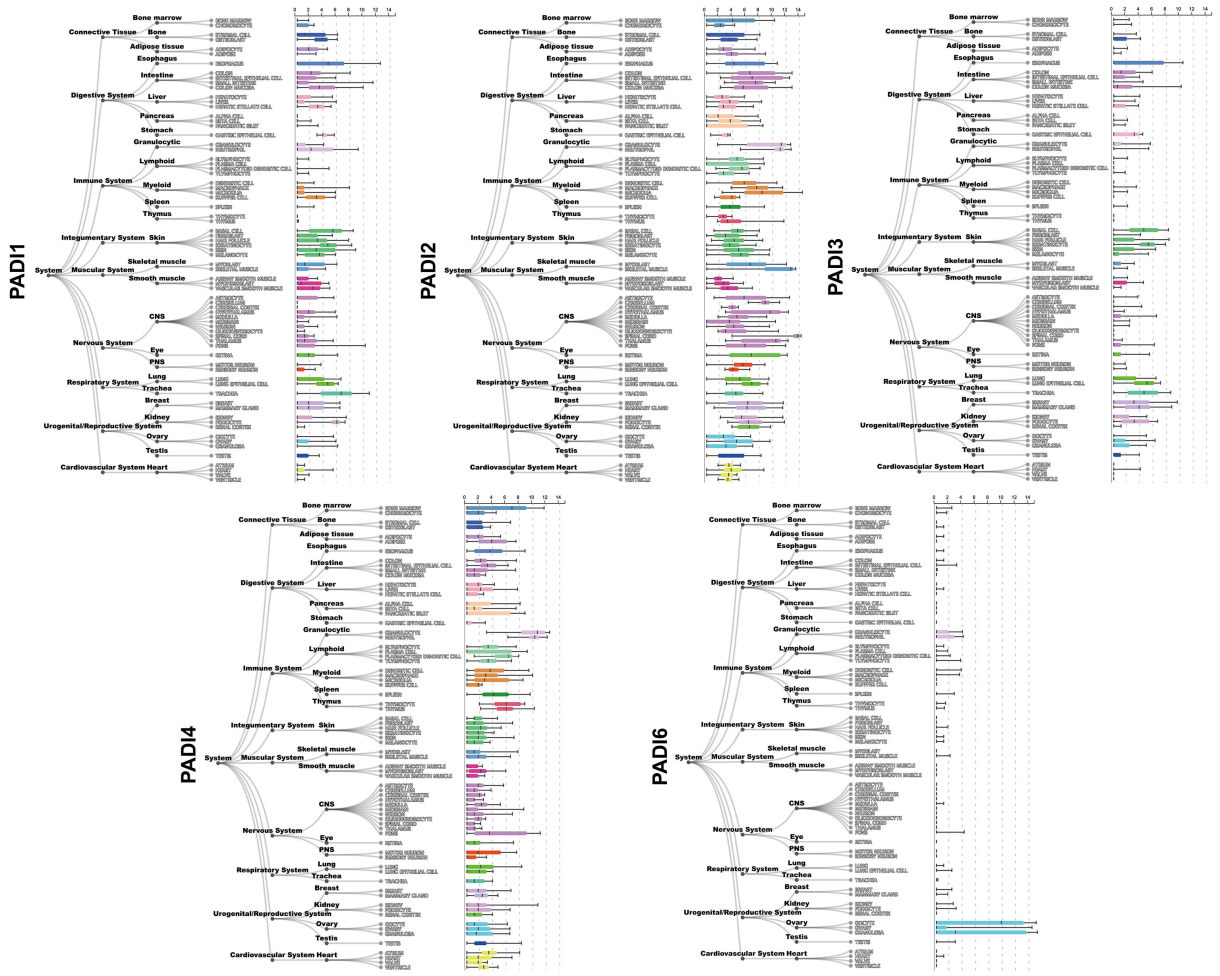
Gene expression of *PADI1*~*PADI4* in different human tissues. Gene expression data is provided by ARCHS^4^ database (6) (https://maayanlab.cloud/archs4/index.html).

The distribution of the five PAD isoforms varies across tissues and organs. PAD1 is primarily expressed in the epidermis (4) and the reproductive system (5). PAD2 is the most widespread isoform and is existed in many tissues and organs, including the central nervous system, intestines, skin, skeletal muscle, eyes, lungs, breast, ovary, and immune cells (**Figure 2**). PAD3 is abundant in human stem cells, hair follicles, and the epidermis (7). PAD4 is highly tissue-specific and abundant in the bone marrow and immune cells, especially in neutrophils (**Figure 2**). PAD6, the only catalytically inactive isoform, chiefly exists in the female reproductive system (8, 9). Among the five isoenzymes, PAD2 and PAD4 have been the most extensively investigated. Both PAD2 and PAD4 can be translocated into the nucleus, indicating their ability to regulate genes in several ways (10). PAD4 possesses a canonical nuclear localization signal (NLS) and was once thought to be the only PAD isoform that could enter the nucleus (11); however, subsequent studies indicated that PAD2 could also be able to citrullinate histone H3 in the nucleus (12, 13).

One of the research hot spots for PAD4 in neutrophils is NETosis. Activated neutrophils capture and eliminate pathogens by unleashing neutrophil extracellular traps (NETs) containing depolymerized chromatin and intracellular granule proteins. NETosis refers to the death of neutrophils associated with NETs formation, a process different from apoptosis and necrosis. It consists of depolymerized chromatin as a mesh scaffold, housing several components, including citrullinated histone 3 (CitH3), neutrophil elastase (NE), myeloperoxidase (MPO), cathepsin G, and other cytoplasmic proteins (14). A strong CitH3 specificity in NETosis facilitates the evaluation of the level of NETs (15). NETosis is a double-edged sword—it not only eliminates pathogens but also damages tissue given its cytotoxicity and proinflammatory properties (16). As an essential part of innate immunity, it influences the development of many diseases, including vasculitis, autoimmune diseases, sepsis, periodontitis, tumors, and tumor cell metastasis (17).

## 2 Physiological functions of PADs

The physiological functions of PADs depend on the tissues wherein they are expressed. By interacting with specific substrates, PADs-mediated citrullination performs various functions. Primary substrates along with the distribution and functions of PADs are briefly summarized in **Table 1**. Given the distribution and substrate specificity, the functions of PAD1 and PAD3 are typically in the maintenance of hair follicles and skin structures (18). PAD1 and PAD3 are essential in the process of constitutive epidermal autophagy, a critical stage in keratinization (4). Apoptosis-inducing factor, a mitochondrial protein, is citrullinated by PAD3 for apoptosis induction in neural stem cells during neuronal development (19). PAD1 and PAD6 are crucial in embryonic development, and although PAD6 is catalytically inactive, its absence terminates embryonic development (20–22).

**Table 1:** Primary Substrates, distribution, and physiological functions of PADs.

PADs	Primary Substrates	Main Distribution	Physiological Function
PAD1	Keratins, Profilaggrin	Keratinocytes, epidermis, hair follicles, reproductive system	Sustains epidermal integrity and supports embryonic development
PAD2	Histone H3, Vimentin MBP, Fibulin-5	Widely distributed (female tissue, gastrointestinal tract, myeloid leukocytes, neural cells, etc.)	Diverse functions (gene regulation, neurodevelopment, immune regulation, formation of pulmonary elastic fiber, etc.)
PAD3	Trichohyalin, S100A3, Apoptosis-inducing factor	Hair follicles, mammary glands, neural stem cells	Maintaining structural stability of hair follicles and regulating apoptosis of neural stem cells during neurodevelopment
PAD4	Histone H3, Collagen, Chemokines, von Willebrand factor cleaving protease, ADAMTS13	Bone marrow, neutrophils, monocytes, macrophages, eosinophils, mast cells	Gene regulation, formation of extracellular traps (ETs) against pathogenic microbes and thrombosis
PAD6	—	Oocytes	Embryonic development

Owing to the broad expression profile of PAD2, its functional range is diverse. PAD2 in the central nervous system is associated with plasticity for neurological development in children but its excessive activation may result in multiple sclerosis (23). PAD2 is necessary for oligodendrocyte differentiation, myelin formation, and motor functions (24). Moreover, PAD2 is a transcriptional activator of the estrogen receptor (13). Protein expression of PAD2 is initiated in monocytes during their transformation into macrophages, suggesting its relevance during macrophage differentiation (25). PAD2 also influences the differentiation of CD4+ T cells and the transition from B cells to plasma cells, indicating its immunomodulatory function (26, 27). A recent study showed that PAD2-mediated fibulin citrullination facilitated the generation of pulmonary elastic fibers. Citrullination protected fibulin from degradation and subsequent elastic-derived inactivation. Ablation of PAD2 in mice caused age-dependent emphysema (28).

PAD4 is recognized as a crucial enzyme in NETosis, a neutrophil response to eliminate pathogens in innate immunity (17). Recently, its role in thrombosis was confirmed. Citrullination of von Willebrand factor (VWF)-cleaving protease and ADAMTS13, two natural substrates of PAD4, results in their deactivation, thereby retarding VWF degradation and ultimately facilitating thrombosis (29). Moreover, healthy neutrophils spontaneously release PAD2 and PAD4, responsible for extracellular citrullination under physiological conditions (30). However, the specific functions and mechanisms of this appear to be unknown.

In the digestive system, as shown in **Figure 2**, PAD2 is highly expressed. Indeed, 121 citrullinated proteins have been found in the gut mucosa of healthy individuals, including cytoskeletal proteins, extracellular proteins, membrane proteins, and anti-citrullinated protein antibodies (ACPAs) (31). However, the physiological functions of PAD2 and most citrullinated proteins in the gastrointestinal (GI) tract remain unclear. Given the negligible expression of PAD1 and PAD3 in the digestive system, their physiological roles herein have been rarely studied. PAD4, however, is abundant in neutrophils that are tightly associated with intrinsic immunity, inevitably occupying a place in the physiological functioning of the digestive system. Undoubtedly, PAD4-mediated NETs protect against pathogenic microorganisms like bacteria, fungi, viruses, and amoebas in the digestive tract. DNA reticulum can capture and trap pathogens, while granular proteases like NE and MPO possess antibacterial ability (17). Proper activation of NETs by PAD4 is crucial for maintaining homeostasis of the GI tract. Disrupting the balance, its physiologically protective function can adversely promote inflammation and tissue damage.

## 3 NETosis and NETs

The mechanisms underlying NETosis occurrence remain unclear but it is generally believed that there are three main types–suicidal (lytic), non-suicidal (vital), and mitochondrial (32). Suicidal NETosis can be triggered by PMA or ligands bound to the Fc receptor, in turn rupturing neutrophil membranes and releasing depolymerized chromatin, histones, along with granule proteins into the extracellular space. Vital NETosis is induced by microbial infection recognized by TLR2 or complement receptors, as well as platelet activation by lipopolysaccharide (LPS) binding to TLR4. As cells are alive, DNA fragments and histones after chromatin depolymerization are released through exocytosis. Under specific stimulation (GM-CSF + LPS/complement factor 5a), neutrophils release mitochondrial DNA into extracellular compartments resulting in mitochondrial NETs formation (32). However, studies on mitochondrial NETs are scarce. Both suicidal and non-suicidal NETosis can activate PAD4, which in turn translocates to the nucleus causing histone citrullination and resulting in chromatin depolymerization, along with its extracellular release. However, PAD4 may not be required in all instances of NETs formation (33), recent studies show that selectively inhibiting or deleting PAD2 also reduces the formation of NETs in neutrophils under certain conditions (34, 35), indicating that PAD2 also serves a role in NETs formation.

Owing to the development of mass spectrometry (MS), citrullinated proteins have been identified. Several proteomic studies confirm the presence of citrullinated proteins in NETs, including MPO, NE, histone H1/3/4, catalase, S100A12, S100A8, S100A9, azurocidin, myeloid cell nuclear differentiation antigen, vimentin, actin, high mobility group protein B1/2, etc. (36–38). These studies, however, report relatively different amounts and categories of citrullinated proteins, which may be due to differences in detection techniques, algorithms, and methods for stimulating NETs formation. Notably, all the above studies chose peripheral blood neutrophils for NETs proteomic analyses. Further research is required to determine whether it can mimic neutrophils’ behavior in inflammatory tissue areas. Moreover, citrullinated proteins identified by proteomics need to be validated by downstream molecular experiments, which are currently not feasible for all, given the lack of specific antibodies.

Along with NETosis, macrophages, eosinophils, and mast cells also release DNA extracellular traps (ETs), resulting in METosis (39), EETosis (40), and MCETosis (41). Some researchers have referred to this type of cell death, whereby ETs are released, as ETosis (10). Like NETosis, ETosis also involves the elimination of pathogenic microorganisms and the induction of inflammation (42, 43). In particular, METosis in response to TNF-alpha stimulation has been reported in the macrophage cell line RAW264.7, with PAD2 rather than PAD4 playing a significant role in the process (44). At present, PAD4 may be essential for ETosis but the above finding also suggests the role of PAD2. Based on existing studies, whether PAD2 holds an equivalent function as PAD4 in Etosis, remains further investigation. In short, PAD2 and PAD4 most likely play valuable roles in human immune-, inflammation-, and tumor-associated diseases of the digestive system.

## 4 Pathological functions of PADs in the digestive system

**Figure 3:**
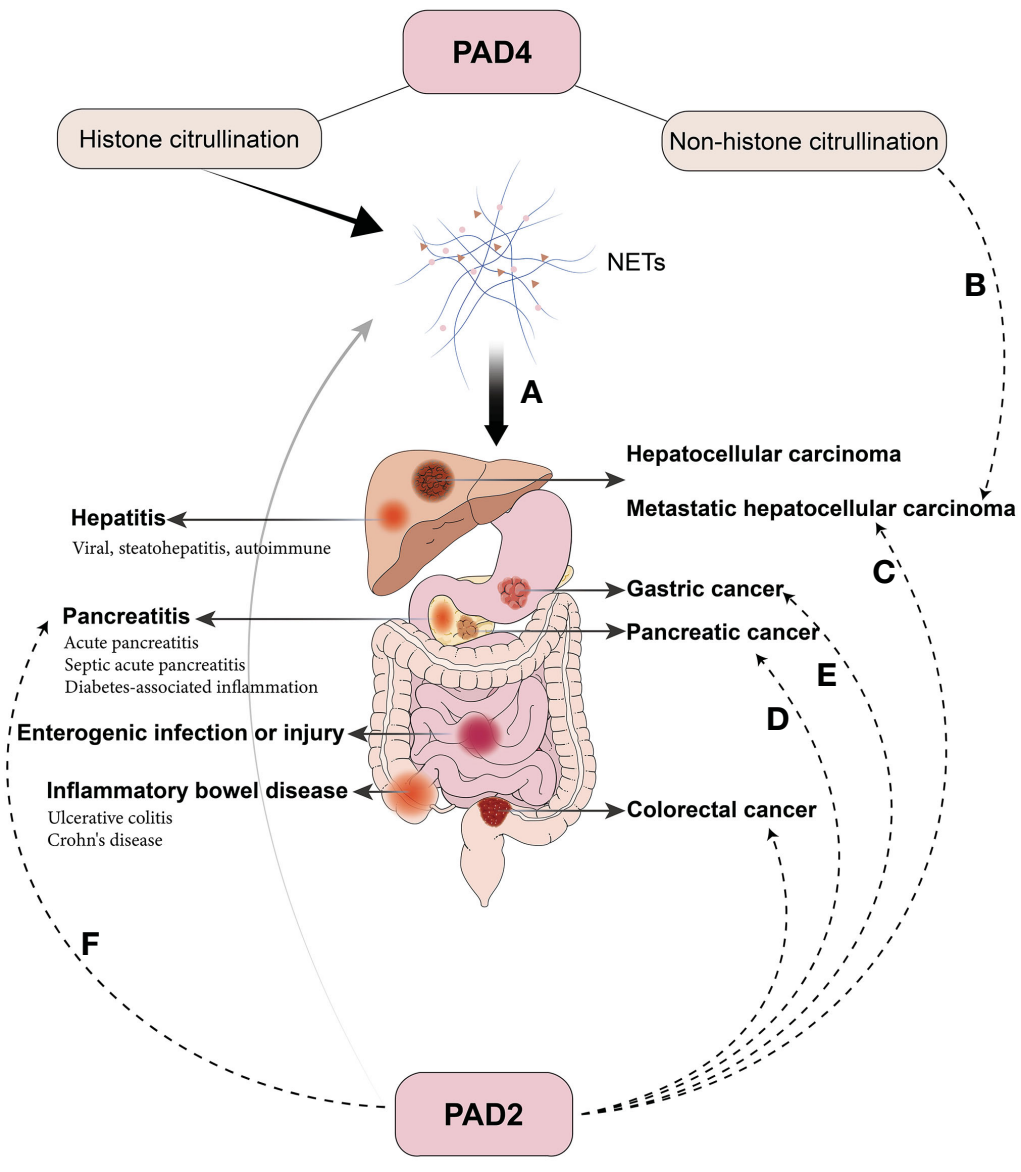
Pathological Roles of PAD2/4 in digestive diseases. **(A)** PAD4-mediated histone citrullination results in NETs and contributes to various inflammatory and oncological diseases in the digestive system. **(B)** Citrullination of extracellular non-histone proteins mediated by PAD4 promotes liver metastasis in colorectal cancer. **(C)** PAD2 promotes colorectal cancer cell invasion and liver metastasis by METs and citrullination of β-catenin. **(D)** PAD2 is highly expressed in several pancreatic cancer cells, and selective inhibitors suppress the invasion and regulate the secretion of anti- or pro-oncogenic miRNA in extracellular vesicles. **(E)** PAD2 inhibition hinders cell growth and migration in gastric cancer. **(F)** PAD2 is elevated in septic AP and correlates with CitH3 and patient prognosis.

The adequate activity of PADs and appropriate citrullination levels are essential in maintaining normal physiological functions. However, under pathological conditions, excessive PAD activation and hypercitrullination disrupt the balance, preventing proteins from performing their functions due to structural modifications. This leads to a range of problems, including dysfunction, inflammatory activation, autoantibody formation, and impaired gene regulation, which contribute to multiple diseases of many systems. Overactivated PAD4 in neutrophils also contributes to the excessive generation of NETs, transforming NETs from innate protective factors to destructive ones. Based on the extensive study of PAD2 and PAD4, the subsequent discussion is directed toward PAD2/4 and excessive NETs formation in the digestive system and is summarized in **Figure 3**.

PADs are a family of calcium-dependent enzymes. They require a high Ca^2+^ concentration for activation *in vitro* (~mM); however, intracellular Ca^2+^ concentrations in the physiological state are low (~μM) and the specific mechanism of their activation *in vivo* appears unclear (12). PADs are equipped with four to six Ca^2+^-binding sites essential for their enzymatic activities. A high concentration of Ca^2+^ leads to sequential conformational changes in PADs, inducing catalytically active sites and increasing their activity by more than 10,000-fold (45). Ca^2+^ influx (46), emission of intracellular Ca^2+^ pools (12), and synergistic activation by other substances (47) are the possible reasons for intracellular PAD activation. Moreover, increased bicarbonate concentration, redox conditions, and hypoxia also modulate the activity of PAD (48). Endoplasmic reticulum (ER) stress can contribute to the release of intracellular Ca^2+^ pools, and ROS can enhance suicidal NETosis (32). Multiple digestive diseases, including IBD, AP, and nonalcoholic steatohepatitis (NASH), reportedly elevate ER stress and ROS levels, possibly contributing to elevated PAD4 activation and NETs levels (49–51).

In inflammatory diseases, neutrophil-derived NETs play a dominant role. In addition to the release of intracellular Ca^2+^ pools due to ER stress, perforin, membrane attack complexes (MAC), bacterial calcium ionophores, and pore-forming toxins due to inflammatory states or pathogen attack may also contribute to neutrophil Ca^2+^ influx and PAD4 activation (52). NETs can exacerbate inflammatory responses through a range of mechanisms, including acting as damage-associated molecular patterns (DAMPs), activating complements, and triggering inflammasomes (53). In autoimmune-related diseases, citrullinated proteins in NETs create neoepitopes that can be recognized by autoimmune factors, thus triggering autoantibody generation. Abnormal hypercitrullination mediated by PADs may also contribute to the disease initiation. A subset of PAD4 autoantibodies, or PAD3/4 autoantibodies, due to antigenic cross-reactivity, enhance PAD4 activity by decreasing the threshold of Ca^2+^ required for PAD4 activation (54), thereby contributing to hypercitrullination. In tumors, both NETs produced by neutrophils and PADs expressed by tumor cells have been implicated. On the one hand, NETs are associated with tumor inflammation and immunity. NETs, although trapping tumor cells, inevitably isolate the tumor cells and tumor-killing cells from immune contact. Furthermore, circulating tumor cells tend to be captured by NETs in distant organs, thus creating conditions for metastasis. On the other hand, the high expression of PAD in tumor cells may affect tumor cell proliferation, migration, and invasion by interfering with the citrullination of signaling pathway proteins (55, 56). In the following sections, we elaborate on the pathological functions of PADs and NETs, from inflammatory to oncological diseases in the digestive system.

### 4.1 Inflammatory bowel disease

PAD4-mediated NETs may play a role in promoting the progression of inflammatory bowel disease (IBD). Levels of NETs-associated products and expression of PAD4 increased in the intestinal tissues of IBD patients and mice with DSS-induced colitis. The content of circulating extracellular DNA also increased in DSS-induced colitis mice. MPO, NE, and citrullinated histone levels were elevated in the intestinal tissues of ulcerative colitis (UC) patients with significant co-localization (57). Components of NETs were found in intestinal biopsy tissues of pediatric IBD patients (58). The level of PAD4 expression correlated significantly with the histopathologic grade, degree of anatomic lesions, treatment ineffectiveness, and presence of radical surgery. UC patients with high histopathologic grade activity, total colon involvement, strong/moderate PAD4 expression levels, and ineffectiveness to conventional pharmacologic regimens were suitable for radical surgery (59). Similarly, in a mouse model of TNBS-induced Crohn’s disease (CD), the production of NETs increased markedly; inhibition of PAD4 effectively alleviated colonic inflammation (60). Clinically, NETs-related proteins are overexpressed in the inflammatory colon of UC patients compared to CD patients and normal subjects (61), suggesting a more prominent role for NETs mediated by PAD4 in UC than CD.

Recently, Dragoni et al. summarized the role of NETs and citrullination in IBD (62). They mainly focused on the damaging effects of NETs and citrullination on the intestinal tract. Nevertheless, NETs may also play a protective role in the pathogenesis of IBD, which is hard to neglect. Bennike et al. (63) found that elevated levels of NETosis could protect by facilitating pathogen elimination. During the clearance of molecular patterns associated with tissue damage, the required cytokines depend on enzymatic regulation in NETs (64). Gutiérrez et al. suggested that NETs reduced intestinal mucosal permeability and lowered the risk of bacterial translocation in IBD patients. Inhibition of NETs by degrading DNA resulted in increased intestinal mucosal permeability in mouse colitis models (65). Moreover, recent research also showed that the existence of PAD4 and the ability to generate NETs promoted immunothrombosis and attenuated colonic bleeding in injured mucosa of DSS-induced colitis mice (66). In another review, the benefits of NETs in IBD have also been described (67).

ACPAs target citrullinated proteins and peptides and act as highly specific biomarkers of rheumatoid arthritis (RA), contributing to disease pathogenesis and outcome (68). It is widely believed that ACPAs are disease-specific predictors, and they are hardly ever detected in diseases other than RA. In IBD, ACPAs are rarely reported. One study found that some patients with IBD may have elevated ACPAs, which level is not sufficient to meet the diagnostic threshold for RA (69). As the increase of ACPAs can occur long before the onset of RA (70), this cross-sectional study (69) lacks long-term follow-up data and cannot guarantee whether these ACPA-positive patients are prone to develop RA in the future.

### 4.2 Pancreatitis

Acute pancreatitis (AP) typically begins with aseptic inflammation and may progress to local or systemic infections and even sepsis in severe cases. NETs levels in both pancreas and plasma were significantly elevated in AP. PAD4 knockout attenuated pancreatic inflammation by reducing NETs formation in animal experiments. PAD4 not only functions as an aggravating factor after pancreatitis development but also triggers its development. Bicarbonate or calcium carbonate components of pancreatic fluid elicit PAD4-mediated NETs production. High-density NETs can form aggregates that block pancreatic ducts, leading to pancreatic inflammation, while PAD4 deficiency prevents disease progression (71). Recently, Li et al. summarized the advances in basic research on NETs in AP and the detection of NETs in different AP models (72). Apart from PAD4 and NETs, PAD2 also seems to be involved in AP. Septic AP activated PAD2, PAD4 and CitH3, leading to an increase in NETs. There was a significant increase in CitH3 levels among cases of infectious AP compared to healthy volunteers or noninfectious AP. Moreover, CitH3 showed positive correlations with PAD2, PAD4, dsDNA, and sequential organ failure assessment scores, indicating that CitH3 mediated by PAD2/4 was a potential diagnostic and prognostic indicator for septic AP (73). Further, circulating CitH3 derived from NETs evoked the expression of proinflammatory factors, disrupting endothelial cell function *via* cytotoxicity and oxidative stress, thus contributing to multi-organ damage (74).

Type 1 diabetes (T1D), closely related to islets in pancreas, is an autoimmune disease with a remarkable immune correlation with the destruction of β cells. Evidence suggests that neutrophil infiltration and the formation of NETs are observed before the onset of T1D (75). A recent study also found high expression of PAD2 mRNA in islet cells, instead of neutrophils, most likely occurring at an earlier stage before inflammatory infiltration. PAD2-mediated citrullination may induce autoantibody generation and consequently increase susceptibility to T1D (76), suggesting a possibly greater role of PAD2 in pancreatic autoimmune diseases, unlike neutrophil-mediated acute inflammation. Alternatively, NETs formation due to intestinal barrier dysfunction and leakage of intestinal microbe induced differentiation of T cells to autoimmune Th1/Th17 cells and promoted inflammatory responses in islets, thus establishing a microbe-gut-pancreas-T1D connection (77).

### 4.3 Hepatic diseases

In recent decades, the incidence of nonalcoholic fatty liver disease (NAFLD) has dramatically increased and become the most common chronic liver disease worldwide (78). Nonalcoholic steatohepatitis (NASH) is an inflammatory subtype of NAFLD that can progress over time to cirrhosis hepatocellular cancer and end-stage liver disease. It is associated with metabolic disorders including obesity, type 2 diabetes and hyperlipidemia. In both serum and tissues of NASH patients, NETs were significantly increased. Additionally, animal models showed that the presence of NETs occurred throughout the disease despite an increase and a subsequent decrease in neutrophil levels. In mice that used DNase to eradicate NETs or in PAD4^-/-^ mice, the liver showed milder inflammation. Further *in vitro* analysis showed that free fatty acids in NASH stimulated the generation of NETs in neutrophils, similar to the effect of LPS (79).

The hepatitis B virus (HBV) is a common virus implicated in chronic hepatitis. It is reportedly associated with PAD4-mediated NETs formation. NETs decrease in a chronic state of HBV infection, whereas HBV suppresses NETs release by regulating ROS reduction and autophagy (80). In acute inflammatory conditions, including HBV-related acute-on-chronic liver failure (ACLF), elevated circulating neutrophil counts and ratios are sensitive prognostic indicators of severity and outcomes (81, 82). Neutrophils in ACLF have an increased ability of NETosis but impaired phagocytosis capacity, more prominent in patients with poor prognoses (83). In autoimmune hepatitis (AIH), PAD4-mediated NETs facilitate the externalization of autoantigens causing liver damage. Low-density granulocytes (LDGs), a neutrophil subtype with pro-inflammatory features, are significantly elevated in patients with AIH. LDGs closely correlate with elevated levels of NETs and indicators of liver fibrosis, pointing to the degree of PAD4 activation across different neutrophil subtypes and its potential clinical application (84).

### 4.4 Infection-related diseases and organ injuries in the digestive system

NETs have been implicated in many infection-related digestive diseases. It is impossible to ignore the role NETs play in enterogenic infections. NETs formation can be stimulated by microorganisms in the gut, subsequently exerting antimicrobial effects by limiting the spread of bacteria and locally providing high levels of antimicrobial protection (85). Recent evidence demonstrates the key role of NETs in removing specific bacteria like *Citrobacter rodentium* and limiting the systemic dispersal of bacteria in necrotizing enterocolitis (86). To avoid capture by NETs, pathogens have evolved several escape mechanisms. *Pseudomonas aeruginosa* can secrete DNase to degrade NETs, and thus, protect itself from NETs-mediated oxidative damage by increasing the synthesis of *arn* or spermidine (87). Similarly, *Vibrio cholerae* can rapidly degrade DNA reticulation by producing extracellular nucleases, thereby avoiding the capture and killing effects of NETs (88). These escape mechanisms allow bacteria to accomplish invasion by exploiting the damaging effects of NETs on the intestinal barrier.

Organ injuries like intestinal ischemia-reperfusion injury often appear after the restoration of blood flow in acute intestinal ischemic conditions like trauma, shock, intestinal transplantation, and mesenteric artery embolization. Bacterial translocation, endotoxin release, and inflammatory storms can cause damage to multiple organs, resulting in a systemic inflammatory response or even multi-organ failure. NETs exacerbate intestinal inflammation and destroy the intestinal epithelial barrier, which has been confirmed in a rat model of intestinal ischemia-reperfusion injury. A recent study by Zhan et al. demonstrated that NETs could aggravate organ damage induced by intestinal ischemia-reperfusion injury (89), while DNase I treatment markedly alleviated intestinal injury, suggestive of an unfavorable effect of NETs (90). The aggregation of NETs during infection and sepsis is widely recognized. In a mouse model of polymicrobial sepsis, NETs played a protective role during early immune responses (91). However, increasing evidence suggests that elevated NETs during sepsis may damage the intestinal tract. For example, NETs were reported to impair intestinal barrier functions through ER stress (92). The impairment of NETs in the gut has been demonstrated in a rat model of LPS-induced sepsis (93). Meanwhile, NETs reportedly injure the intestinal tract during trauma-hemorrhagic shock and early intravenous administration of tranexamic acid could be a therapeutic strategy against intestinal barrier dysfunction by inhibiting NETs formation (94).

Briefly, in acute inflammatory diseases of the digestive system, PAD4-mediated NETs play an important role given the high involvement of neutrophils. In the early stages of inflammation, NETs function as protective factors to help clear pathogenic microorganisms; however, when pathogens evolve escape mechanisms, the organism may be forced to produce more NETs to defend itself, and the accumulation of NETs results in excessive inflammatory responses that are damaging to the relevant organs. Herein, increasing the degradation of NETs may be an effective measure to prevent further exacerbation of organ inflammation. Additionally, PAD2-mediated citrullination and neoantigen epitopes in autoimmune-related diseases of the digestive system cannot be ignored. Although few studies have been conducted, findings on islet inflammation and IBD, suggest an association with abnormally citrullinated proteins, making it worthy of further investigation.

### 4.5 Cancers of the digestive system

At present, cancers of the digestive system contribute to the highest mortality rate than any other organ system in humans (95). Accumulating evidence from both basic and clinical studies implies that PAD2 and PAD4 serve critical roles in cancer onset and progression. In esophageal, gastric, colonic, rectal, hepatic and pancreatic cancers, PAD2 and PAD4 are reported to be abnormally expressed. Li et al. (96) showed that in Chinese patients with liver, esophageal, and colon cancers, the expression of PAD2 in the blood and tumor tissues was higher than in normal samples. However, a study from Japan and another from Spain showed significantly lower expression of PAD2 in colorectal cancer tissues compared to normal or adjacent tissues (55). The above findings indicate that the role of PAD2 may be variable across digestive system tumors. Chang et al. (97) found a high PAD4 expression in various tumor tissues, including gastric adenocarcinoma, esophageal squamous carcinoma, lung adenocarcinoma, hepatocellular carcinoma, and breast cancer. Zhang et al. (98) showed that PAD4-related NETs gene scores were risk factors in multiple cancers, and high scores were associated with poor outcomes. Notably, there are two primary sources of PADs in tumors: (1) PAD4-mediated NETs produced by tumor-associated neutrophils and (2) tumor cells with high PAD2/4 expression. It seems inappropriate to consider the functions performed by these two sources of PADs in tandem. NETs mostly affect tumors at the extracellular level, while PAD2/4 expressed by tumor cells may affect themselves through intracellular mechanisms.

Among all tumors, gastric cancer is the fifth most prevalent and the fourth deadliest type (95). PAD4-mediated NETs in the peripheral blood of gastric cancer patients were associated with tumor progression and metastasis, which may be related to the enhanced epithelial-mesenchymal transition (EMT). Patients with advanced stages showed abnormally activated neutrophil PAD4 and were more susceptible to NETosis (99). Similarly, another study reported that abdominal infection after radical gastric cancer surgery led to a massive number of NETs, promoting the proliferation and metastasis of gastric cancer through the TGF-β signaling pathway and EMT (100). Although the above findings appear to support that NETs facilitate tumor progression and metastasis, the elevated number of NETs may be a result, just an accompanying phenomenon during tumor progression, or possibly only reflects the inflammation status in the tumor. Based on the existing evidence, it is too early to conclude the role of NETs in gastric cancer. Interference with PAD4 expression significantly inhibited the proliferation and invasion of gastric cancer cells, SGC-7901 and AGS, *in vitro (* 101), suggesting that PAD4 may also function in gastric cancer cells independent of NETs, which requires further investigation. In addition to PAD4, *in vitro* experiments also revealed that inhibition of PAD2 expression in gastric cancer cells, MKN-45, could hinder cell growth and migration (100), indicating PAD2’s involvement in gastric cancer. Still, high-quality evidence is needed to determine whether PAD2/4 is a friend or a foe in this context.

Given its insidious onset, pancreatic cancer (PC) is often at an advanced stage when first detected. The sensitivity of existing markers for the diagnosis of early-stage PC is insufficient, therefore, the identification of new diagnostic features has been a research hot spot in the field. PC-associated hypercoagulability is linked with PAD4-mediated NETs formation, increasing the risk of thrombosis and mortality (102). Clinical studies show that tumor-infiltrating neutrophils and their production of NETs might serve as a prognostic factor for PC independent of the TMN staging system (103). Mechanistically, NETs promote pancreatic cell migration and growth by activating the IL-1β/EGFR/EKR pathway and EMT (104). In pancreatic ductal adenocarcinoma, IL-17 recruits neutrophils and promotes the formation of NETs, resulting in the exclusion of CD8+ T cells from the tumor. Blocking of IL-17 increases its sensitivity to immune checkpoint inhibitors, suggesting that IL-17 and PAD4 are potential treatment targets in PC (105). The inhibitor of growth 4 (ING4), a non-histone substrate of PAD4, enhances the transcriptional activity of p53 by binding to it through its nuclear localization signal (NLS) region. PAD4 predominantly citrullinated ING4 in its NLS region, thereby disrupting the interaction between ING4 and p53 (106). According to a recent study conducted on PC cells, PAD2 and PAD3 were highly expressed in two cell lines. Selective PAD2 or PAD3 inhibitors suppressed PC cell invasion and regulated the secretion of anti- or pro-oncogenic miRNA in extracellular vesicles (107). It suggests that in addition to PAD4 and NETs, PAD2 and PAD3 in PC should not be neglected, and further research is needed to compare the roles of different isoforms in PC.

Colorectal cancer (CRC) ranks third in incidence and second in mortality among malignant tumors (95). In CRC patients, PAD4-mediated NETs are elevated in tumor tissues and peripheral circulation, which are also associated with venous thrombosis (108–110). Specifically, NETs are predominant in tumor centers and invasive CRC fronts, indicating their involvement in tumor cell metastasis (111). *In vitro*, NETs activated the EMT, as evidenced by increased expression of vimentin and fibronectin but decreased levels of epithelial cell adhesion molecule and E-cadherin (111). According to Yazdani et al., NETs accelerated mitochondrial synthesis *in vitro*, whereas PAD4^-/-^ mice showed decreased tumor mitochondrial density along with significant reductions in the mitochondrial biosynthesis proteins, PGC-1α, TFAM and NRF-1 (112). Interestingly, CRC cells release PAD4 into the extracellular matrix *via* extracellular vesicles, thus promoting collagen citrullination and EMT (113). Additionally, PAD2-mediated METs might be an independent prognostic factor in CRC patients. Subsequent experiments *in vitro* also demonstrated that METs contributed to CRC cell invasion, which in turn, enhanced METs generation. A specific inhibitor of PAD2 suppressed METs generation and restrained liver metastasis (114). Meanwhile, PAD2 was proven to inhibit the growth of colon cancer cells SW480 and HCT116 by repressing the Wnt/β-catenin pathway through citrullination of β-catenin (55, 56). Nucleophosmin (NPM), a nuclear protein catalyzed by PAD2, plays an essential role in cellular metabolisms including ribosome biogenesis, mRNA processing and chromatin remodeling. PAD2 can citrullinate NPM at site 277, which can be targeted and cleared by CD4+ T cells to exert antitumor effects. It is unlike the PAD4 citrullination of arginine 197 in NPM, leading to the translocation of NPM from the nucleolus to the cytoplasm (115). The above findings imply that PAD2 and PAD4 have a non-negligible role in the invasion and metastasis of CRC cells. Not only can they citrullinate histones in NETs but they can also directly citrullinate non-histone proteins; however, the exact underlying mechanism remains to be elucidated.

In hepatocellular carcinoma (HCC), elevated levels of NETs have been observed in both patients and mice (97, 116). Liu et al. (116) found that LPS facilitated NETs formation *via* TLR4, further promoting hepatic steatosis and HCC in alcohol-fed mice. Animal experiments revealed that PAD4 knockdown significantly reduced the number of hepatic tumor growths in the STAM model (79). A subsequent study found that NETs promoted Treg differentiation to suppress tumor immunosurveillance, thus contributing to carcinogenesis (117). In addition, PAD4-mediated NETs also contribute essentially to hepatic metastasis. NETs are abundant in liver metastases of breast and colon cancers; moreover, serum NETs can predict the onset of early liver metastases in breast cancer patients. NETs not only capture tumor cells but also bind to CCD25 on the tumor cell surface, activating the ILK-β-parvin pathway and promoting tumor proliferation (118). Moreover, PAD4 is essential for the citrullination of the extracellular matrix for liver metastases in CRC. It can be secreted by metastatic focal tumor cells *via* extracellular vesicles, and inhibiting vesicle secretion decreases citrullination and reduces the burden of liver metastases (113).

In summary, PADs function through more complex mechanisms in the development of digestive tumors than in inflammatory diseases. On the one hand, inflammation is a characteristic of tumors, and the killing effect of NETs components like NE on tumors is undeniable. However, growing evidence suggests that NETs can promote tumor progression and are associated with disease prognoses. Given that pathogenic bacteria have evolved escape mechanisms to resist NETs and even use them to complete invasion, do tumor cells develop relevant immune escape mechanisms against NETs or even use NETs to accomplish progression and metastasis? Addressing this question necessitates in-depth mechanistic studies. On the other hand, digestive system tumors may also express some PADs (PAD2/3/4); the mechanism regulating the high expression of PADs and its association with tumor cell escape from NET killing warrants further investigation. What is the role of PADs and NETs in the transition from inflammatory disease to carcinoma (e.g., IBD to CRC, chronic hepatitis to liver cancer)? Several such issues remain to be addressed in future studies.

## 5 Therapeutic strategy and prospects

At present, promising therapeutic strategies for PADs against inflammatory or oncological diseases of the digestive system focus on the following aspects: (1) PAD-specific or pan-inhibitors; (2) DNases that facilitate decomposing of NETs, and (3) other drugs that curb the formation of NETs.

However, effective physiological inhibitors of PAD are lacking while chemically synthesized small-molecule inhibitors (like Cl-amidine and BB-Cl-amidine) are potent in basic experiments. Cl-amidine can reduce clinical parameters and inflammation in TNBS-induced and DSS-induced colitis (60). Cl-amidine attenuates islet inflammation (119) and decreases inflammatory factors in AP (120). However, these compounds can suppress the activity of multiple PADs simultaneously, and the results cannot exclude the impact of other PAD isoforms. For example, given the widespread expression of PAD2, cells highly expressing PAD4 also simultaneously express PAD2. Inhibition of PAD4 using PAD pan-inhibitors may lead to conclusions that include the potential effects of PAD2. Therefore, specific inhibitors for different PADs are of significance for subsequent studies. Recently, the emergence of novel selective inhibitors for PAD2 (AFM-30a) and PAD4 (GSK199, GSK484) have offered the possibility of unraveling the diverse pathophysiological functions for PAD2 and PAD4 (3, 121, 122), and their efficacy and safety need to be assessed in further studies.

DNase is one of the few NETs-degrading drugs in the clinical trial stage, but its utilization remains limited. Although it has not yet been used for digestive diseases, some researchers have pioneered the use of DNase by inhalation in COVID-19 patients under severe conditions and found significant improvements in patient oxygenation (123). However, there are several limitations to the use of DNase, including its short half-life, the need for multiple doses, and susceptibility to degradation by endogenous inhibitors (124). Targeted therapy is a research hot spot but is mainly limited to preclinical stages. Adenoviral vector-mediated DNase I reduces liver metastasis in CRC mice (125), and novel nanomaterials combined with regional light irradiation result in localized DNase release, enhanced immunotherapy, and inhibition of distant metastasis in CRC (124). DNase I not only physically degrades the DNA reticulum of NETs but also reduces neutrophil infiltration and NETs formation by attenuating TLR9 signaling (126) and integrin α M expression (127). Additionally, DNase I enhanced CD8+ T-cell infiltration in liver metastasis, associated with a better prognosis (125).

Other chemicals that affect NET formation exist but are presently confined to basic experimental stages. For example, NETs and NADPH/ROS are closely associated, and the ROS inhibitor, N-acetylcysteine can suppress PMA-induced NETosis in neutrophils (128, 129). Some antioxidants like vitamin C can suppress ROS-dependent NET production. Given the importance of citrullinated proteins in disease progression, therapeutic ACPA (tACPA) seems promising. In mice, to prevent a multitude of PAD4-induced diseases, including IBD, tACAPs are effective (130). Furthermore, other agents like vitamin D (131), metformin (132), and probiotics like *Lactobacillus rhamnosus* GG (133) have also been reported to inhibit NETs formation.

## 6 Conclusion

PADs are isoenzymes with tissue distributional specificity. The preference of PADs for substrates may be related to their structural and tissue specificity (48). Even for the same substrate, the arginine catalytic sites of PAD2 and PAD4 may differ, resulting in varied immunogenic responses, thus exerting diverse or even opposite functional effects (115). Due to high expression in immune cells, PAD4/2-mediated ETosis or ETs plays an essential role in immunity and have been extensively investigated in inflammatory and oncological diseases. Growing evidence suggests that proteins other than histones can be citrullinated by PAD2/4 and serve essential roles in the development of gastrointestinal tumors. Currently, inflammatory diseases of the digestive system primarily emphasize PAD4-mediated NETs, and the citrullination of proteins other than histones has been scarcely studied. Apart from this, PAD2 is abundantly present in the GI tract but its involvement in gastrointestinal inflammatory diseases is largely unknown and requires further investigation. Previous studies have mostly focused on the harmful effects of NETs and the development of diseases. A review of the role of NETs in the intestine by Chen et al. was published in 2021 (134). They mainly concentrate on the harmful effects of NETs on intestinal diseases, slighting the protective role of NETs in inflammation, such as promoting immune thrombosis to reduce colonic bleeding. Additionally, the authors discussed the role of NETs in promoting the progression of colorectal cancer while did not focus on the killing ability of NETs against tumor cells (135). The function of NETs as a double-edged sword needs to be investigated in more detail to answer the following: apart from the immune escape from NET-killing ability, are other mechanisms involved in the switching of NETs from protective to deleterious factors? What is the role of PADs and NETs in the transition from inflammation to carcinoma? Regarding therapeutic prospects, complete inhibition of PADs or ETs is not the best solution given the enhanced susceptibility to bacterial infection (136). Several specific inhibitors of PADs as well as precision-targeted drugs that promote the breakdown of NETs are being investigated by integrative material scientists but maintaining the physiological viability of PADs without their overactivation remains a great concern that needs resolution in future studies.

## Author contributions

Y-HS: Conceptualization and writing the original draft. Z-JW: Data Curation and editing. LK: Review & Editing. Z-XH: Visualization. S-BZ: Investigation. XF: Review & Editing. Z-SL: Supervision. S-LW: Review and funding acquisition. YB: Conceptualization, supervision, Funding acquisition. All authors contributed to the article and approved the submitted version.
